# Host genetic factors associated with the range limit of a European hantavirus

**DOI:** 10.1111/mec.16211

**Published:** 2021-10-21

**Authors:** Moritz Saxenhofer, Anton Labutin, Thomas A. White, Gerald Heckel

**Affiliations:** ^1^ Institute of Ecology and Evolution University of Bern Bern Switzerland; ^2^ Swiss Institute of Bioinformatics, Quartier Sorge – Bâtiment Génopode Lausanne Switzerland

**Keywords:** genome‐wide association study, genomic admixture, host‐pathogen co‐evolution, hybrid zone, *Microtus arvalis*, parasite

## Abstract

The natural host ranges of many viruses are restricted to very specific taxa. Little is known about the molecular barriers between species that lead to the establishment of this restriction or generally prevent virus emergence in new hosts. Here, we identify genomic polymorphisms in a natural rodent host associated with a strong genetic barrier to the transmission of European Tula orthohantavirus (TULV). We analysed the very abrupt spatial transition between two major phylogenetic clades in TULV across the comparatively much wider natural hybrid zone between evolutionary lineages of their reservoir host, the common vole (*Microtus arvalis*). Genomic scans of 79,225 single nucleotide polymorphisms (SNPs) in 323 TULV‐infected host individuals detected 30 SNPs that were consistently associated with the TULV clades CEN.S or EST.S in two replicate sampling transects. Focusing the analysis on 199 voles with evidence of genomic admixture at the individual level (0.1–0.9) supported statistical significance for all 30 loci. Host genomic variation at these SNPs explained up to 37.6% of clade‐specific TULV infections. Genes in the vicinity of associated SNPs include *SAHH*, *ITCH* and two members of the *Syngr* gene family, which are involved in functions related to immune response or membrane transport. This study demonstrates the relevance of natural hybrid zones as systems not only for studying processes of evolutionary divergence and speciation, but also for the detection of evolving genetic barriers for specialized parasites.

## 1 INTRODUCTION

The evolution of viruses and their respective hosts is usually linked by various dependencies. Patterns of co‐evolution can range from highly antagonistic evolutionary arms‐races (Daugherty & Malik, 2012) to commensal or even mutualistic relationships (Roossinck, 2011), which in turn impose varying degrees of selective constraints on both the virus and the host (Hall et al., 2011). In the case of infectious diseases, viruses are predominantly under selective pressure to develop and maintain infection pathways to their hosts, as well as coping with their host's immune response (Pybus & Rambaut, 2009). Viruses generally face a tradeoff in fitness between specializing to maximize efficiency for a single host (specialists) and infecting multiple host species (generalists; Ciota & Kramer, 2010; Deardorff et al., 2011). In both cases the capability of infecting additional host species is a major opportunity for a virus to increase its infection range (Ciota & Kramer, 2010).

Such opportunities, however, may be limited as virus spillover infections often lead to evolutionary dead ends and transmissions are confined in the new host (Mollentze et al., 2020; Webby et al., 2004). Transmissions to new hosts that allow the virus to proliferate and continue spreading are overall infrequent and mediated by a variety of factors, most importantly the degree of relatedness between the hosts and the frequency of host contacts (Luis et al., 2015; Parrish et al., 2008; Streicker et al., 2010). In recent decades, cross‐species transmission, particularly of RNA viruses, has resulted in a large number of disease emergences in humans and livestock, representing a major issue for health systems and economies (Hu et al., 2020; Jones et al., 2008; Lloyd‐Smith et al., 2009; Mayer et al., 2017). Understanding the patterns of viral diversity in reservoir species and the identification of factors that affect cross‐species virus emergence have become the focus of surveillance and prevention programmes (Lam et al., 2020; Olival et al., 2017).

The constitution of species barriers defining the infection range for a virus has been analysed mostly in the context of zoonotic disease emergence (Longdon et al., 2014; Olival et al., 2017; Plowright et al., 2017; Sironi et al., 2015). Phylogenetic analyses of viruses and their corresponding hosts have suggested that genetic factors play a primary role in determining the infection range of viruses (Faria et al., 2013; Longdon et al., 2014; Streicker et al., 2010). Overcoming a species barrier and establishing an efficient transmission chain in a new host may require multiple adaptive changes in the viral genome, representing a major evolutionary challenge (Kuiken et al., 2006; Parrish et al., 2008; Reperant et al., 2012; Simmonds et al., 2019).

The natural infection ranges of some viruses are restricted to closely related species (Streicker et al., 2010) or even to evolutionary lineages of the same host species (Drewes et al., 2017; Gryseels et al., 2017; Saxenhofer et al., 2019). Experimental studies comparing the infection efficiency of viruses in cells of different host species have allowed the identification of single host genes limiting the species range that certain viruses can infect, such as coronaviruses (van Doremalen et al., 2014), HIV‐1 (Stremlau et al., 2004) or influenza A virus (Long et al., 2016). This suggests that relatively few intrinsic differences between otherwise genetically similar hosts can mediate strong barriers to virus emergence. The order of evolutionary events and the relative contribution of adaptive vs. neutral changes is difficult to determine in natural virus–host systems but it is possible that divergence of key genes involved in virus–host interactions could occur early in the process of host speciation (Saxenhofer et al., 2019). Still, it remains generally unclear how a host's genetic factors shape the selective environment of associated viruses and determine their infection range in natural populations.

Here we investigate intrinsic genetic factors delimiting the distribution ranges of Tula orthohantavirus (TULV) among evolutionary lineages of its reservoir host, the common vole (*Microtus arvalis*), using a genome‐wide association study (GWAS) approach. In hantaviruses (family Hantaviridae; formerly Bunyaviridae), several immunity‐related host genes have been associated with virus replication efficiency and persistence in their rodent reservoirs (see Charbonnel et al., 2014 for review). However, the focus was mostly on hantavirus infections in humans (Liu et al., 2009; Mäkelä et al., 2001; Martínez‐Valdebenito et al., 2019; Müller et al., 2019; Mustonen et al., 1996; Wang et al., 2009) with relatively little information available on genes that regulate hantavirus resistance in reservoir species (Guivier, Galan, Salvador, et al., 2010; Rohfritsch et al., 2018). The consequences of candidate host genes for hantaviral infection ranges in nature remain mostly unclear (Dubois et al., 2017; Rohfritsch et al., 2018).

TULV is a single‐stranded RNA virus with a three‐segmented genome that is horizontally transmitted and causes asymptomatic chronic infections in the rodent reservoir host (Forbes et al., 2018; Vaheri et al., 2013). In Europe, the large‐scale geographical distribution of highly diverged phylogenetic clades in TULV is partially associated with the distribution of morphologically cryptic evolutionary lineages in *M*. *arvalis* (Heckel et al., 2005; Schmidt et al., 2016). A detailed study of an intraspecific hybrid zone in the common vole demonstrated a very tight spatial association between the host lineages Central and Eastern and the virus clades Central South (TULV‐CEN.S) and Eastern South (TULV‐EST.S; Saxenhofer et al., 2019). A strong evolutionary barrier to effective TULV transmission in the hybrid zone operates at distances that individual voles can travel in very short time (Hahne et al., 2011; Saxenhofer et al., 2019; Schweizer et al., 2007) and where gene flow between host lineages is ongoing (Beysard & Heckel, 2014). Deep genomic divergence (17% sequence difference) and the absence of recombination or reassortment between TULV‐CEN.S and TULV‐EST.S indicate that these viral clades exceed the stage of speciation of the host lineages (Saxenhofer et al., 2019). The zone of contact between these TULV clades contains no physical barriers, such as rivers or changes in altitude, which might impede host movements or TULV transmission and is thus likely to be driven by intrinsic genetic factors that probably arose in the host lineages before secondary contact after post‐glacial recolonization formed the hybrid zone (see Saxenhofer et al., 2019).

In this study, we take advantage of this natural system to interrogate the host genome for genetic polymorphisms associated with the sharp and probably selectively maintained distribution range limit of TULV clades that behave effectively as distinct viral species (see Saxenhofer et al., 2019). We focused our GWAS on the zone of natural admixture where the cosegregation of genetic polymorphisms distinguishing host lineages with the TULV clades is partially broken up by many generations of hybridization after secondary contact (Beysard & Heckel, 2014). Employing replicate sampling transects served to limit the potential of detecting geographically restricted associations and enabled the identification of genomic polymorphisms and genes in *M*. *arvalis* that may contribute to confining the range of this European hantavirus.

## 2 METHODS

### 2.1 Sample collection

**FIGURE 1:**
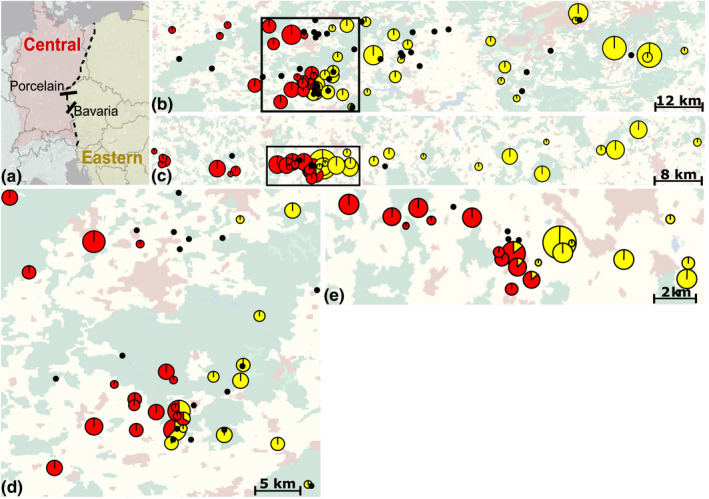
Sampling locations of common voles (*Microtus arvalis*) and Tula orthohantavirus. (a) Map of Central Europe with the approximate position of the hybrid zone (dashed line) between the Central (red) and Eastern (yellow) evolutionary lineages in *M*. *arvalis* and the location of the Porcelain and Bavaria sampling transects (full lines). (b,c) Complete view of the Porcelain (b) and Bavaria (c) transects containing the contacts between TULV‐CEN.S (red) and TULV‐EST.S (yellow) clades. Each circle represents a sampling location with its area proportional to the number of infected individuals. Black dots represent locations where no infected individuals were found. (d, e) Detailed view of the contact zone between TULV‐CEN.S and TULV‐EST.S for the Porcelain (d) and Bavaria (e) transect. The background in (b)–(e) shows settlements (red), forests (green), water bodies (blue) and cultivated land (yellow)

We sampled 547 common voles mostly from the direct contact area between the TULV‐CEN.S and the TULV‐EST.S clades and combined them for our analyses with TULV‐infected samples available from Saxenhofer et al. (2019) (Table 1 and Table S1). Sampling was performed in two replicate transects (Porcelain and Bavaria; ~130 km apart) across the hybrid zone between the Central and Eastern common vole evolutionary lineages (Figure 1). Common voles were trapped using snap traps and stored at −20°C immediately after collection. Rodent trapping was performed after ethical evaluation and approval by the Bernese cantonal commission on animal experimentation under permits BE‐90/10 and BE‐33/14. Host DNA was extracted according to a standard phenol–chloroform protocol. DNA concentration was measured for each sample using the Qubit dsDNA BR Assay Kit (Invitrogen) and DNA quality was evaluated on an agarose gel.

**TABLE 1:** Number of common vole individuals from two sampling transects across the hybrid zone screened for Tula orthohantavirus

	TULV‐CEN.S	TULV‐EST.S	Uninfected	Total
Porcelain transect	62 (53)	101 (80)	687 (0)	850 (133)
Bavaria transect	98 (89)	123 (101)	479 (0)	700 (190)
Both transects	160 (142)	224 (181)	1166 (0)	1550 (323)

Note

The number of voles infected with the TULV‐CEN.S or TULV‐EST.S clades is given. The number of individuals included in the GWAS data set is indicated in parentheses.

### 2.2 TULV screening and clade assignment

Molecular screening for TULV infection was performed on the 238 adult voles of at least 20 g from 32 sampling sites among the 547 newly sampled individuals in the Porcelain and Bavaria transects. Voles of less than 20 g bodyweight were not assessed for TULV infection as young individuals are typically protected by maternal antibodies (Kallio et al., 2006; Schmidt et al., 2021). TULV infection was detected by amplifying a 540‐nucleotide fragment of the nucleocapsid gene on the small TULV genome segment (S‐segment) following the RT‐PCR (reverse transcriptase PCR) assay described in Essbauer et al. (2006). RNA was extracted from lung tissue, and S‐segment fragments of TULV‐positive samples were sequenced as described by Schmidt et al. (2016) and Saxenhofer et al. (2019). A phylogenetic analysis was performed with mrbayes version 3.2.6 (Ronquist et al., 2012) on the CIPRES platform (Miller et al., 2010) to assign TULV sequences to major evolutionary clades using reference sequences from Schmidt et al. (2016) and Saxenhofer et al. (2019) (Table S2). Metropolis‐coupled Markov chain Monte Carlo (MCMC) sampling was performed for 10^7^ generations in four independent runs comprising four chains. We implemented reversible‐jump sampling over the entire general time‐reversible substitution model space (Huelsenbeck et al., 2004) and samples were recorded every 10^3^ generations after discarding a burn‐in fraction of 25%.

### 2.3 Host genotyping

Genotyping by Sequencing (GBS; Elshire et al., 2011) was carried out for all TULV‐infected common voles in both transects (Figure 1) on the Illumina NextSeq platform at Cornell University. Restriction enzymes *Pst*I and *Msp*I were used to generate the libraries in 96‐well plates. Single nucleotide polymorphisms (SNPs) were identified and individuals genotyped simultaneously using the gbs version 2 pipeline (part of the tassel 5 software; Glaubitz et al., 2014), using a chromosome‐level *Microtus arvalis* genome assembly as the reference sequence (Gouy, A., Wang, X., Neuenschwander, S., Schmid, E., Heckel, G. Excoffier, L.; unpublished data). Default parameters were used for calling genotypes, except that a minimum of five reads were required to identify a unique tag. Only bi‐allelic SNPs were retained, and genotypes were only called if individuals had a read depth for that locus of at least five. After SNP calling, individuals with more than 50% of loci with missing data were removed. Then, loci were filtered out if they had a minor allele frequency (MAF) of less than 5%, more than 20% missing data or observed heterozygosity greater than 50%, which may indicate multiple paralogues merged into single loci (White et al., 2013). Three loci with complex indels were also removed. This filtering resulted in a data set of 323 TULV‐infected individuals genotyped at 79,225 SNPs, which corresponds to an approximate density of 1 SNP per 50 kb of vole genome.

### 2.4 Host population structure

Previous population structure analyses based on microsatellite markers in the hybrid zone within the Bavaria and Porcelain transects have already shown genetic admixture between the common vole lineages in these regions, supporting *K* = 2 genetic clusters (Beysard & Heckel, 2014; Saxenhofer et al., 2019). To examine the extent to which these patterns of genetic admixture were also present in the infected individuals of our genomic data set, we analysed the genetic structure in the hybrid zone with the admixture software (Alexander et al., 2009; Zhou et al., 2011). The analyses were performed for both transects separately using only TULV‐infected individuals. We used initial cross‐validation implemented in admixture for *K* = 1–10 which supported *K* = 2 for both transects. We then performed admixture bootstrapping with 1000 replicates for each transect to establish genetic cluster membership of each infected individual.

### 2.5 Genome‐wide association analysis

A Genome‐wide Efficient Mixed Model Association (gemma) analysis was conducted with version 0.98.1 of the software (Zhou & Stephens, 2012). As gemma requires a data set without missing data, we used ld‐knni as implemented in tassel 5 (Glaubitz et al., 2014) for the imputation of missing SNPs based on the mean of the 10 closest related neighbours. Neighbours were determined by the 30 physically closest SNPs for each missing site and individual. Imputation was performed independently for the whole data set, as well as for each transect separately, and any SNPs still possessing missing data or a MAF of less than 5% were removed. Correcting for population structure within the data set was initially performed using covariates (principal components 1 and 2) and/or relatedness matrix (calculated via gemma version 0.98.1). However, this led to an overcorrection of the GWAS results due to the transition of virus phenotypes (TULV clades) within the host hybrid zone (Saxenhofer et al., 2019). Alternatively, we made use of the replicated nature of our data set to identify genetic polymorphisms in the host that are less dependent on the local population structure in each transect. We ran gemma’s linear model on default parameters for the whole data set of 323 infected individuals, as well as separately for the Bavaria transect (190 infected individuals) and Porcelain transect (133 infected individuals). Association strength of individual SNPs was estimated with gemma by calculating the Wald test *p*‐value (Wald, 1945) and corrected for multiple testing with the Bonferroni method (Bonferroni, 1936). Only SNPs which were significantly (*p* < .05) associated with clade‐specific TULV infections across all three GWAS (Full data set, Bavaria transect only, Porcelain transect only) were considered for further analyses. To estimate the effect size of loci associated with TULV clades across both transects, a probit model was fitted on the genotype information of individual SNPs using the glm function followed by calculating Nagelkerke's *R*‐squared (Nagelkerke, 1991) implemented in the fmsb package in R.

### 2.6 Candidate genes

We analysed a flanking region of 100 kb up‐ and downstream (based on Brodie, Azaria, & Ofran, 2016; Laurie et al. 2007) of every significant SNP for genes that could serve as candidate genes for restricting the TULV infection range. We identified all genes in these flanking regions based on the presence of start or stop codons within the window around the significant SNP and determined their identity by using blast (Altschul et al., 1990) to detect their closest homologue in the well‐annotated mouse reference genome. We performed an enrichment analysis of all candidate genes using the panther 16.0 classification system as part of the Gene Ontology (GO) Consortium online resource (Ashburner et al., 2000; Consortium, 2018; Mi et al., 2018). We used Fisher's exact test to find significantly enriched GO terms for both the biological processes and molecular functions of our candidate genes and corrected for multiple testing with the Bonferroni method (Bonferroni, 1936). For all genes with a GO term relevant for virus infection (i.e., related to immune response or membrane/vesicle transport), we examined further if there was published evidence that either they were involved in virus‐related pathways, or a family member of a gene involved these pathways.

## 3 RESULTS

### 3.1 TULV infections of common voles in the sampling area

The screening of 238 new common voles from 32 locations in the zone of admixture identified 78 TULV‐infected individuals (33%), adding to the 306 infected individuals detected in the wider area by Saxenhofer et al. (2019) (Table 1 and Table S1; GenBank accession nos.: OK317919–OK317991 and OK356661–OK356665). Phylogenetic inference of TULV sequences from the region together with reference sequences assigned 160 sequences to the TULV‐CEN.S and 224 to the TULV‐EST.S clade (Figure S1). TULV‐CEN.S infections were only detected in the western parts and TULV‐EST.S only in the eastern parts of both transects (Figure 1). Common voles collected from the same population were typically infected with virus strains from the same TULV clade except for six locations (three in each transect) where TULV sequences from both clades were detected.

### 3.2 Genetic admixture within the host hybrid zone

**FIGURE 2:**
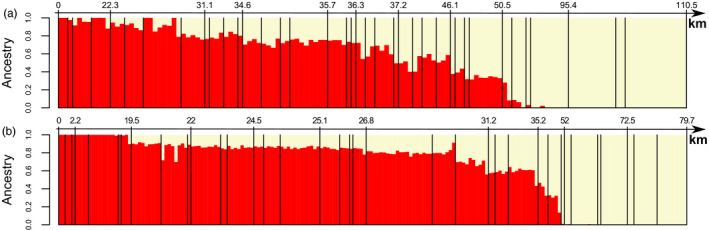
Genetic structure of common voles infected with Tula orthohantavirus from the Porcelain (a) and Bavaria (b) transects. Each vertical bar represents the assignment of an individual to the Central (red) and Eastern (yellow) evolutionary lineage determined by the admixture software based on 79,225 genetic markers. Black vertical lines separate individuals from different sampling locations. Distances from the western end of the transect are given in kilometres (km)

GBS data were successfully obtained for 323 TULV‐infected individuals sampled close to the putative zone of admixture in the transects. This included 190 individuals from the Bavaria transect and 133 individuals from the Porcelain transect (Table 1). The analysis of ancestry based on 79,225 SNPs demonstrated the presence of two major genetic clusters representing the Central and Eastern common vole evolutionary lineages (Beysard & Heckel, 2014; Heckel et al., 2005; Lischer et al., 2013) in both transects, with a clear transition between mostly pure populations at the transect ends (Figure 2). Of 323 infected individuals, 199 showed evidence of admixture with cluster memberships of 0.1–0.9 to either cluster.

### 3.3 Genome‐wide association across two replicate transects

**FIGURE 3:**
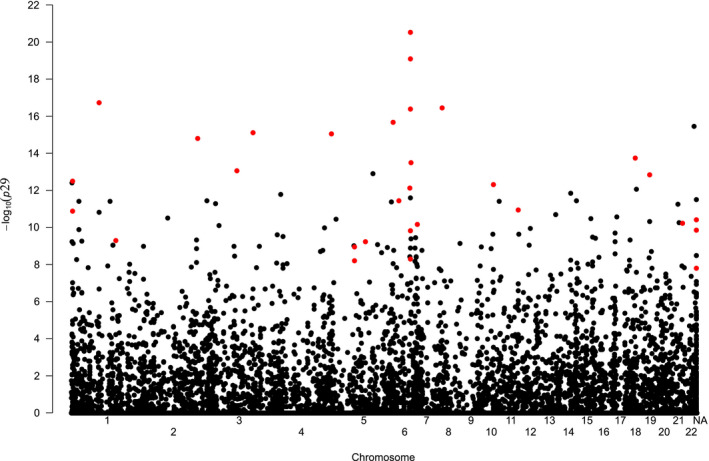
Results of genome‐wide association analyses of 323 common voles infected with Tula orthohantavirus (TULV) plotted for 38,715 SNP markers shared between alternative GWAS data sets. Red dots indicate the 30 SNPs which were significantly associated (*p* < .05) with the infection by a specific TULV clade in each of four complementary data sets (all 323 individuals; Bavaria transect: 190 individuals; Porcelain transect: 133 individuals; no‐imputation: 165 individuals; see text for details). The *y*‐axis indicates the *p*‐value after correction for multiple testing with the Bonferroni method

Filtering of SNPs with a MAF of less than 5% and imputation of missing data for the GWAS provided a total of 60,471 SNPs for the full data set containing both transects, 57,461 SNPs for the Bavaria transect and 64,490 SNPs for the Porcelain transect. In total, 38,715 SNPs were shared between all three data sets. Among those, we found a total of 32 SNPs that showed a significant association with TULV clades in separate GWAS of the three individual data sets (Figure 3). We observed a particularly strong association on Chromosome 6 across a region of ~1 MB, containing five of the 32 significant SNPs and the two SNPs with the lowest *p*‐values.

To assess the potential impact of imputation on the GWAS results, we removed any of the 323 individuals that had any missing data among the 32 significant SNPs, which resulted in 165 remaining individuals. This analysis showed that two of the 32 initially significant SNPs no longer passed the significance threshold for association with the TULV clades. To further assess the possible impact of spatial autocorrelation of vole lineages and virus clades, we ran a separate GWAS including only the 199 admixed individuals with cluster membership between 0.1 and 0.9. All of the 30 significant SNPs were detected again in this conservative analysis and no new SNPs were found to be significantly associated.

### 3.4 Immune‐system related candidate genes

The examination of a 100‐kb up‐ and downstream flanking region for each of the 30 SNPs significantly associated with TULV‐CEN.S or TULV‐EST.S infection revealed a total of 105 candidate genes with homologues in the *Mus musculus* genome (Table S3). We detected no significant enrichment of GO‐terms among these genes (all *p* > .3). Among our candidate genes we identified nine with published evidence of involvement in viral response or infection pathways or that are a family member of such a gene (Table 2). The flanking regions of the three SNPs with the lowest *p*‐values in all GWAS contained four genes with known relevance in viral infection pathways: *S*‐adenosyl homocysteinase (*SAHH*, SNP *p* = 2.88 × 10^−21^), E3 ubiquitin‐protein ligase Itchy (*ITCH*, SNP *p* = 7.66 × 10^−20^) and the two genes Synaptogyrin‐3 and Tuberous Sclerosis Complex 2 near the third locus (*Syngr03*/*TSC2*, SNP *p* = 1.8 × 10^−17^; Table 2). Both *SAHH* and *ITCH* are located within the highly associated region on chromosome 6 between the two top scoring SNPs that are ~150 kb distant from each other. *Syngr03* corresponds to a region on chromosome 1, with the associated SNP located in the 4^th^ exon of *Syngr03*. *TSC2* corresponds to a region of 33 kb that is 56 kb downstream of the very same SNP. For readability and because *Syngr03* is closer to the associated SNP, we refer to the *Syngr03*/*TSC2* locus in the following simply as the *Syngr03* locus. Fitting a probit regression model demonstrated an effect size of 37.6% for the *SAHH* locus, 37.1% for the *ITCH* locus and 32.8% for the *Syngr03* locus, which represents the proportion of clade‐specific TULV infections explained by the associated host genotype (Table 2).

**TABLE 2:** Candidate genes potentially involved in restricting the spatial range of TULV CEN.S and EST.S clades

Ensembl ID	Chromosome	Position	Gene	Distance	*p*‐Value	Function	*R* ^2^
ENSMUST00000054607	6	58206488	SAHH	85,231	2.88E‐21	Methyltransferase regulation	.376
ENSMUST00000029126	6	58585061	ITCH	85,038	7.66E‐20	Ubiquination of target proteins	.371
ENSMUST00000007236	1	87846372	SNG3	0	1.86E‐17	Membrane trafficking and transport	.328
ENSMUST00000097373	1	87846372	TSC2	55,654	1.86E‐17	Anabolic metabolism regulation	.328
ENSMUST00000007216	3	145514324	AP2M1	29,732	7.56E‐16	Clathrin‐dependent endocytosis	.27
ENSMUST00000109790	6	56882593	ASXL1	58,637	7.13E‐13	DNA and/or histone modification	.277
ENSMUST00000096482	11	43643687	SKP2	39,432	1.11E‐11	Microvesicle membrane formation	.265
ENSMUST00000026649	1	4414266	SNG2	63,136	1.25E‐11	Membrane trafficking and transport	.241
ENSMUST00000068714	5	66704109	SOS1	20,630	1.05E‐09	Ras‐guanine exchange factor	.222

Note

Only genes involved in virus‐related immune response or membrane/vesicle transport are shown. A complete list of all 105 candidate genes is given in Table S2. Chromosome and position show the location of the SNP in the *Microtus arvalis* reference genome, while distance gives the number of base pairs of the candidate gene and the respective SNP. The *p*‐value refers to the GWAS of all 323 infected individuals. The *R* ^2^ column refers to the phenotypic variance in clade‐specific TULV infections explained by the SNP.

### 3.5 Clade‐specific TULV infections and associated host genotypes

**FIGURE 4:**
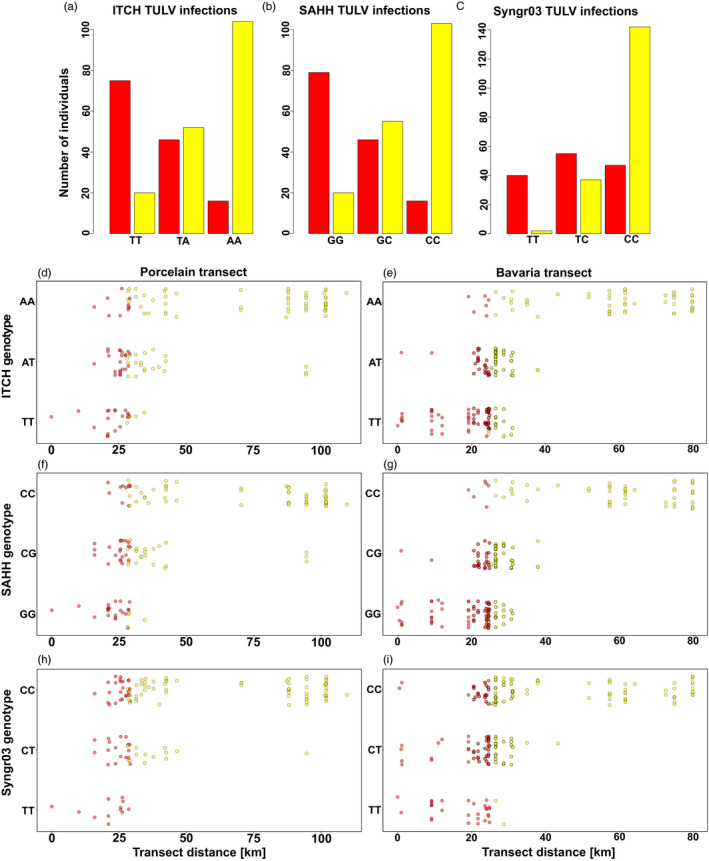
Genotypes at SNPs associated with candidate loci *SAHH* (a), *ITCH* (b) and *Syngr03* (c) and clade‐specific TULV infections. The number of individuals infected with TULV‐CEN.S (red) and TULV‐EST.S (yellow) is given for homozygous Central lineage genotypes, heterozygotes and homozygous Eastern lineage genotypes. (d–i) Spatial distributions of genotypes along the Porcelain (d, f, h) and Bavaria (e, g, i) transects. Vertical jitter was added for each genotype class for better visibility of individual data points

Individual‐level and spatial patterns of the SNPs at the candidate loci emphasize a strong association with infections with TULV‐CEN.S or TULV‐EST.S (Figure 4). For both *SAHH* and *ITCH*, most individuals homozygous for the alleles predominant in the Central or Eastern lineage were infected by the respective virus clade, while heterozygous hosts were infected by the two TULV clades at similar frequencies (Figure 4a,b). In contrast, the homozygous Central genotype at *Syngr03* was more strongly associated with restriction of TULV‐EST.S infection than the Eastern genotype for TULV‐CEN.S (Figure 4c). In the spatial context of the hybrid zone, the allele frequencies at the candidate loci showed a clear transition along both transects (Figure 4d–i). The spatial distribution of homozygous genotypes for the Central and Eastern *SAHH*‐ or *ITCH*‐associated SNPs was highly consistent with the geographical range of TULV‐CEN.S and TULV‐EST.S (Figure 4d–g), thus potentially contributing to the limitations of the nonadapted virus clade. For *Syngr03*, the distribution of the homozygous Central genotype was highly consistent with the range of the TULV‐CEN.S clade. TULV‐EST.S was almost absent from hosts with homozygous Central lineage *Syngr03* genotypes, while the Eastern lineage allele extended far into the range of TULV‐CEN.S (Figure 4h, i).

## 4 DISCUSSION

Our analyses demonstrate very clear spatial patterns of local genomic admixture between common vole lineages and corroborate the extremely abrupt transition between parapatric TULV clades within the hybrid zone. Relatively few SNPs in the rodent host were consistently associated with infection by specific TULV clades across replicate sampling transects, explaining up to 37.6% of the variance. Even at the very fine geographical scale of our analyses, the effects of genetic polymorphisms appear to limit the range of the two TULV clades, providing strong evidence for the evolution of an infection barrier within a host species.

The geographical distribution of pathogens within a host species is impacted by many factors, including landscape features (Guivier et al., 2011) or polymorphisms in resistance against pathogen infections (Alves et al., 2019; Magwire et al., 2012; Rohfritsch et al., 2018; White et al., 2011). Spatially narrow contacts between TULV‐CEN.S and TULV‐EST.S clades (Figure 1c,d) and an absence of potential dispersal barriers, such as rivers or forests (see also Saxenhofer et al., 2019), render a major effect of landscape connectivity (Gryseels et al., 2017), climatic differences (Gloria‐Soria et al., 2017) or other extrinsic factors in the prevention of virus transmission unlikely.

The infection range limits of the TULV clades are probably caused by differences in TULV fitness in the two host lineages because hantavirus infection is largely asymptomatic in common voles and other reservoir hosts (Forbes et al., 2018). The detection of two individuals infected by both TULV clades (Hiltbrunner & Heckel, 2020; Saxenhofer et al., 2019) rules out a full resistance phenotype. Instead, we suggest that host gene polymorphisms that arose in allopatric glacial refugia of the evolutionary lineages of *Microtus arvalis* may act as a partial transmission barrier to the individual TULV clades (see Saxenhofer et al., 2019). Maintenance of a local transmission chain is essential for virus persistence in a host population (Biek & Real, 2010; Forbes et al., 2018; Kuiken et al., 2006). Less efficient virus transmission among individuals of the opposite host lineage (e.g., due to longer transmission intervals) may form part of the barrier for differentially adapted TULV clades (Saxenhofer et al., 2019). The very narrow contact area of TULV‐CEN.S and TULV‐EST.S clades (Figure 1) implies virus transmission primarily among adjacent vole populations (see also Saxenhofer et al., 2017; Schmidt‐Chanasit et al., 2010; Weber de Melo et al., 2015). Direct competition for susceptible hosts among viruses from different clades could only occur in mixed populations or when viruses would be transferred across the clade contact by a dispersing vole host. TULV prevalence in local populations varies between 0% and 45% (Maas et al., 2017; Schmidt et al., 2016, 2021; Schmidt‐Chanasit et al., 2010), which would potentially provide enough noninfected local hosts for transmission across the clade contact. However, the rarity of host populations with both TULV clades and double‐infected individuals (see Saxenhofer et al., 2019) suggests only limited potential for direct within‐host competition between clades to contribute to establishing the range limits.

The GWAS and the spatial context show a strong association between particular SNPs in the host and clade‐specific TULV infections. In principle, a narrow and consistent contact zone of the TULV clades in both transects suggests the involvement of only a single gene of major effect or a small number of tightly linked genes in restricting the infection range of the TULV clades. The transmission barrier might be mediated by a matching‐allele type of virus–host interaction (Dybdahl et al., 2014) where changes in allele frequencies of these host genes along the transects represent a major barrier for transmission of differentially adapted TULV clades (Saxenhofer et al., 2019). Single host factors that restrict the infection range and impede virus emergence have been described so far predominantly between relatively distantly related host species with highly divergent genetic and ecological backgrounds (Long et al., 2016; Stremlau et al., 2004; van Doremalen et al., 2014). In hantaviruses, studies on the interactions with the host immune system have revealed several host factors involved in virus infection, persistence and replication (Charbonnel et al., 2014; Easterbrook et al., 2007; Guivier, Galan, Male, et al., 2010; Martínez‐Valdebenito et al., 2019; Müller et al., 2019). However, infection barriers for hantaviruses have been characterized mostly in the context of human infections (Liu et al., 2009; Mäkelä et al., 2001; Martínez‐Valdebenito et al., 2019; Mustonen et al., 1996; Wang et al., 2009). The rodent host genes identified in this study may present new candidates involved in restricting viral infection ranges and in limiting host shifts of hantaviruses between closely related species.

Among the candidate genes, *SAHH*, *ITCH* and *Syngr03* as well as its close neighbour *TSC2* stand out as particularly promising candidates (Table 2). *SAHH* enables methylation of a variety of both DNA and RNA motifs, which is crucial for the replication of several virus species such as vaccinia virus, yellow fever and vesicular stomatitis (Borchardt et al., 1984; Tseng et al., 1989). *ITCH* is an E3 ubiquitin ligase and a common target for viral hijacking to recruit host proteins necessary for virus development and budding in multiple viruses including Ebola (Han et al., 2016), Herpes (Koshizuka et al., 2018) and Influenza A. *Syngr03* is a member of the Synaptogyrin gene family, which is involved in vesicular transport and endo‐ and exocytosis crucial for virus replication (Sessions et al., 2009; Sun et al., 2016; Walker et al., 2018). Among our candidate genes is also *Syngr02* with a spatial allele distribution resembling that of *Syngr03* (Table 2 and Figure S2). Finally, *TSC2* is involved in anabolic metabolism in cells and a crucial target of the Human Cytomegalovirus UL38 protein, facilitating efficient viral replication (Bai et al., 2015; Moorman et al., 2008). None of these genes has been implicated in hantavirus–host interactions so far, but comparable genomic analyses at similar scale are lacking for other natural systems except for an investigation into the genetics of bank vole tolerance to Puumala hantavirus (PUUV; Rohfritsch et al., 2018).

Further characterization of our candidate genes in the hantavirus context is necessary to identify potential interactions with the investigated TULV clades. Signatures of positive selection on a protein binding signal peptide region of the TULV M‐Segment (Saxenhofer et al., 2019) indicate adaptation towards specific host genes. Many years of research on human‐pathogenic hantaviruses and others have shown the difficulty in identifying key genes or proteins conferring resistance or differences in susceptibility to infection in dead‐end hosts such as humans but also in reservoir hosts (Charbonnel et al., 2014; Martínez‐Valdebenito et al., 2019; Müller et al., 2019; Rohfritsch et al., 2018). The well‐defined ecological, evolutionary and spatial context of this natural system analysed here holds the potential for using even more refined genomic approaches for complementing and prioritizing the list of candidate host factors (see Atkinson et al., 2021; Kwok et al., 2021), which may then feed back into research on pathogenic systems.

Cross‐species transmission of viruses is related to ecological and evolutionary diversity in many mammalian and avian host taxa (Allen et al., 2017; Mollentze & Streicker, 2020), and intrinsic species barriers for viruses may arise mostly as a by‐product of host diversification (Cuypers et al., 2020; de Bellocq et al., 2015; Gryseels et al., 2017; Martinů et al., 2020). In the TULV system, Central and Eastern are the evolutionarily closest lineages in the common vole (Beysard & Heckel, 2014; Lischer et al., 2013; Sutter et al., 2013) and the restrictions in the range of TULV‐CEN.S and TULV‐EST.S have probably arisen only after the host lineages have diverged in allopatry and established secondary contact in the hybrid zone (Saxenhofer et al., 2019). It would thus be interesting to examine the factors and dynamics of the range limits between (hanta‐)virus clades within single host species or lineages further. These could represent transient situations related to range expansion processes of viruses within hosts or be maintained in place by selection related to host polymorphisms (see also Martinů et al., 2020; Theodosopoulos et al., 2019). The TULV system potentially allows a direct test of these alternatives in the future with multiple additional TULV clade contacts in Central Europe, both within and between evolutionary lineages of its rodent host (see Saxenhofer et al., 2019; Schmidt et al., 2016; Schlegel et al., 2012). Studying the genomic barriers to virus transmission across TULV contact zones of different evolutionary divergence analogous to their hosts (Beysard & Heckel, 2014; Beysard et al., 2015) may offer insights into the evolutionary mechanics that drive the divergence of hantaviruses and potentially even the generation of new virus species. For closely related and human‐pathogenic PUUV, many virus clades have been described across Europe and partially associated with particular evolutionary lineages in the bank vole host and the regional absence of the disease in humans (Drewes et al., 2017), but the direct link to genomic polymorphisms in the rodent host has not been established.

## 5 CONCLUSIONS

Extant pathogen populations in animal reservoirs are the most common source of outbreaks of infectious diseases in humans and livestock (Jones et al., 2008; Mollentze & Streicker, 2020). Understanding the factors that affect cross‐species virus emergence is the focus of research and prevention programmes but the combat against outbreaks is often impeded by very limited knowledge about the reservoir hosts (Groseth et al., 2007; Shi et al., 2018). As RNA virus evolution is mostly driven by patterns of co‐evolution and co‐divergence with host taxa (Lin et al., 2012; Mélade et al., 2016; Switzer et al., 2005), combining evolutionary analyses of host and virus divergence directly may allow insights that are difficult to obtain in the nonequilibrium situation of disease outbreaks (Cuypers et al., 2020; Schneider et al., 2021). The explicit consideration of the spatial context of the association of many hantaviruses with their host taxa may be particularly beneficial for clarifying the relationships and succession of events in evolutionary adaptation or host‐species switches (see also de Bellocq et al., 2015,2018; Bennett et al., 2014; Cuypers et al., 2020; Gryseels et al., 2017; Guo et al., 2013; Martinů et al., 2020; Saxenhofer et al., 2017, 2019; Worobey et al., 2010). Our analyses demonstrate that detailed examination of natural hybrid zones between host taxa—or more generally admixture between hosts—has the potential to aid in identifying not only genetic polymorphisms relevant for developing and maintaining species barriers among the hosts but also those loci that contribute to these processes in tightly associated parasites.

## Conflicts of Interest

The authors declare no conflicts of interest.

## AUTHOR CONTRIBUTIONS

G.H. conceptualized the study. M.S., G.H. and T.A.W. performed fieldwork and generated the data for this study. M.S., A.L. and T.A.W. analysed the data. A.L., M.S. and G.H. wrote the original draft. All authors contributed to the reviewing and editing of the final manuscript.

## Supporting information

Supplementary Material

## Data Availability

The Tula virus S‐segment sequence data are openly available in GenBank under accession nos. OK317919–OK317991 and OK356661–OK356665. Raw GBS sequence data are available at the NCBI Sequence Read Archive (SRA) at https://www.ncbi.nlm.nih.gov/bioproject/PRJNA767008. The keyfile for matching GBS reads to individual samples together with the GWAS results are available at the Dryad repository under https://doi.org/10.5061/dryad.5dv41ns6p.
