# Supplementary material for: Host genetic factors associated with the range limit of a European hantavirus
Source: Mol Ecol. 2021 Oct 21;31(1):252–65. doi: 10.1111/mec.16211 (PMC9298007; doi:10.1111/mec.16211)
Supplement: Supplementary file 1 — Supplementary Material [file MEC-31-252-s001.docx]

**Supplemental Information for:**

**Host genetic factors associated with the range limit of a European hantavirus**

Moritz Saxenhofer, Anton Labutin, Thomas A. White, Gerald Heckel


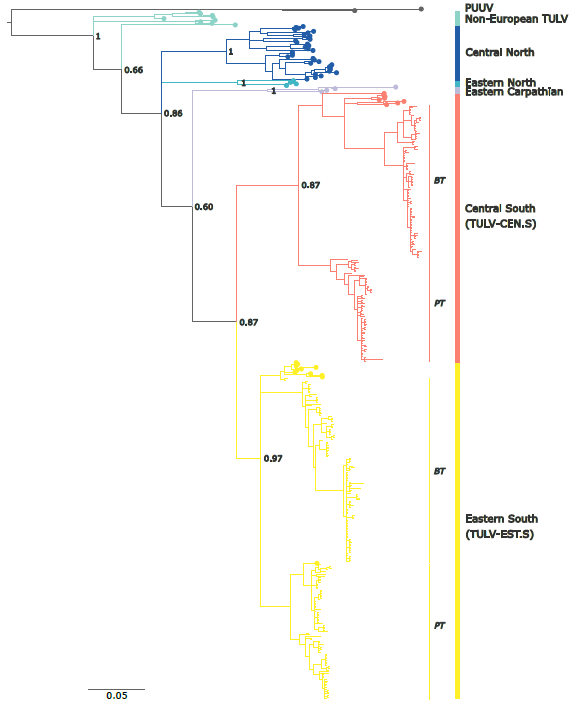


**Figure S1:** Assignment of Tula orthohantavirus (TULV) sequences from the common vole hybrid zone (blunt tips) to phylogenetic clades using published reference sequences (round tips). The origin of TULV sequences from the Porcelain transect (PT) or Bavaria transect (BT) is indicated. Colors indicate major TULV clades and labels include the name of the associated common vole lineage and the geographic location of TULV clades in it (Saxenhofer et al. 2019). The tree represents a maximum clade credibility topology of a Bayesian analysis with posterior probabilities stated for major nodes. Puumala orthohantavirus (PUUV) served as outgroup. See Supplementary Table S2 for reference and outgroup sequence accession numbers.

**Figure S2:** Genotypes at a SNP associated with candidate locus *SYNGR2* and clade specific TULV infections. (A) The number of individuals infected with TULV-CEN.S (red) and TULV-EST.S (yellow) is given for homozygous Central lineage genotypes, heterozygotes and homozygous Eastern lineage genotypes. Spatial distributions of genotypes along the Porcelain (B) and Bavaria (C) transects. Vertical jitter was added for each genotype class for better visibility of individual data points.

**Supplementary Table S1:** Sampling locations in two replicate transects (Bavaria, Porcelain) across the hybrid zone between the Central and Eastern evolutionary lineages of *Microtus arvalis*. Distance of the sampling site from the respective transect start is given in km analogous to Saxenhofer et al. (2019). The columns CEN.S and EST.S show the number of individuals detected with the respective Tula orthohantavirus clade and GWAS reports the number of individuals included in the association analyses.

| **Site** | **Year** | **Transect** | **Latitude** | **Longitude** | **Distance** | **CEN.S** | **EST.S** | **GWAS** |
| --- | --- | --- | --- | --- | --- | --- | --- | --- |
| Arnschwang | 2010 | Bavaria | 49.2762 | 12.795 | 38.89 | 0 | 4 | 4 |
| Arzberg A | 2015 | Porcelain | 50.0455 | 12.1681 | 31.77 | 1 | 0 | 1 |
| As | 2014 | Porcelain | 50.2149 | 12.2166 | 36.59 | 0 | 1 | 1 |
| Baerenfelder | 2016 | Bavaria | 49.1834 | 12.6647 | 25.27 | 3 | 0 | 3 |
| Baerlas | 2014 | Porcelain | 50.1924 | 11.8777 | 14.15 | 1 | 0 | 1 |
| Baernbach | 2016 | Bavaria | 49.1815 | 12.6670 | 25.31 | 5 | 0 | 5 |
| Bernstein A | 2011 | Porcelain | 50.0761 | 12.0624 | 23.72 | 0 | 0 | 0 |
| Birkov | 2010 | Bavaria | 49.5249 | 13.2269 | 80.61 | 0 | 10 | 10 |
| Bochov | 2014 | Porcelain | 50.1565 | 13.0668 | 95.47 | 0 | 10 | 10 |
| Bor | 2013 | Porcelain | 50.1666 | 12.4438 | 51.21 | 0 | 0 | 0 |
| BrennbergA | 2010 | Bavaria | 49.0684 | 12.3916 | 1.87 | 1 | 0 | 1 |
| BrennbergB | 2010 | Bavaria | 49.0753 | 12.3945 | 2.22 | 4 | 0 | 4 |
| Bruckbach | 2016 | Bavaria | 49.0821 | 12.3661 | 1.52 | 1 | 0 | 1 |
| Chudenice | 2010 | Bavaria | 49.4731 | 13.2031 | 75.74 | 0 | 7 | 7 |
| Cichalov | 2014 | Porcelain | 50.1290 | 13.1665 | 102.49 | 0 | 2 | 2 |
| Dietersgruen | 2015 | Porcelain | 50.0776 | 12.1942 | 33.07 | 0 | 2 | 2 |
| Dornhof | 2014 | Porcelain | 50.0268 | 12.2032 | 34.71 | 0 | 4 | 4 |
| Dvorek | 2013 | Porcelain | 50.1433 | 12.4226 | 49.43 | 0 | 0 | 0 |
| Elisenfels | 2015 | Porcelain | 50.0409 | 12.169 | 31.95 | 2 | 0 | 2 |
| Elisenfels Sued | 2016 | Porcelain | 50.0357 | 12.1664 | 31.91 | 0 | 1 | 1 |
| Erkersreuth | 2015 | Porcelain | 50.1936 | 12.1327 | 30.16 | 1 | 0 | 1 |
| Falkenstein | 2010 | Bavaria | 49.1073 | 12.4963 | 10.45 | 9 | 0 | 9 |
| Faschaberg | 2016 | Bavaria | 49.1709 | 12.6262 | 22.14 | 1 | 0 | 1 |
| Fingermuehl | 2015 | Bavaria | 49.1098 | 12.5253 | 12.51 | 1 | 0 | 1 |
| Frantiskovy Lazne | 2013 | Porcelain | 50.1259 | 12.3612 | 44.92 | 0 | 0 | 0 |
| Fridau | 2016 | Porcelain | 50.0196 | 12.1589 | 31.88 | 0 | 2 | 2 |
| Furth im Wald | 2010 | Bavaria | 49.3009 | 12.8601 | 44.34 | 0 | 1 | 1 |
| Garmersreuth | 2015 | Porcelain | 50.0579 | 12.1281 | 28.7 | 2 | 0 | 2 |
| Garmersreuth | 2016 | Porcelain | 50.0579 | 12.1281 | 28.7 | 1 | 0 | 1 |
| German Border | 2015 | Bavaria | 49.3311 | 12.9698 | 52.82 | 0 | 2 | 2 |
| Gredlmuehle | 2016 | Bavaria | 49.1898 | 12.6804 | 26.62 | 0 | 1 | 1 |
| Groppenheim | 2015 | Porcelain | 50.0189 | 12.2480 | 38.03 | 0 | 3 | 3 |
| Gutmanning | 2015 | Bavaria | 49.2031 | 12.6840 | 27.62 | 0 | 19 | 19 |
| Gutmanning | 2016 | Bavaria | 49.2031 | 12.6840 | 27.62 | 0 | 1 | 1 |
| Hayek | 2010 | Bavaria | 49.3735 | 13.0172 | 58.32 | 0 | 8 | 8 |
| Hazlov | 2011 | Porcelain | 50.1547 | 12.2822 | 39.59 | 0 | 0 | 0 |
| Hildbrandsgruen | 2014 | Porcelain | 50.2099 | 11.7339 | 11.67 | 1 | 0 | 1 |
| Hof | 2016 | Bavaria | 49.1998 | 12.6872 | 27.62 | 0 | 7 | 7 |
| Horni Dvory | 2014 | Porcelain | 50.0718 | 12.4065 | 48.27 | 0 | 2 | 2 |
| Horni Lomany | 2013 | Porcelain | 50.1357 | 12.3403 | 43.5 | 0 | 7 | 7 |
| Horni Paseky | 2015 | Porcelain | 50.2228 | 12.2605 | 39.85 | 0 | 3 | 3 |
| Hrebeny | 2013 | Porcelain | 50.2132 | 12.5662 | 60.68 | 0 | 0 | 0 |
| Hundsbach | 2015 | Porcelain | 50.0270 | 12.3222 | 43 | 0 | 1 | 1 |
| Jeronym | 2013 | Porcelain | 50.1007 | 12.7368 | 71.75 | 0 | 3 | 3 |
| Kamenna Osada | 2014 | Porcelain | 50.2405 | 12.1644 | 34.25 | 0 | 0 | 0 |
| Kanicky | 2014 | Bavaria | 49.4606 | 13.1776 | 73.43 | 0 | 2 | 2 |
| Knoebling | 2010 | Bavaria | 49.1645 | 12.6019 | 20.26 | 2 | 0 | 2 |
| Knoebling | 2015 | Bavaria | 49.1645 | 12.6019 | 20.26 | 7 | 0 | 7 |
| KnoeblingB | 2015 | Bavaria | 49.1707 | 12.6195 | 21.72 | 8 | 0 | 8 |
| Korbersdorf | 2015 | Porcelain | 50.0310 | 12.1294 | 29.49 | 1 | 0 | 1 |
| Korbersdorf | 2016 | Porcelain | 50.0310 | 12.1294 | 29.49 | 2 | 0 | 2 |
| Kopanina | 2011 | Porcelain | 50.2049 | 12.458 | 52.92 | 0 | 0 | 0 |
| Kothigenbibersbach | 2015 | Porcelain | 50.0820 | 12.1546 | 30.21 | 0 | 0 | 0 |
| Kothigenbibersbach | 2016 | Porcelain | 50.0820 | 12.1546 | 30.21 | 4 | 0 | 4 |
| Kvetna | 2013 | Porcelain | 50.2069 | 12.5237 | 57.56 | 0 | 0 | 0 |
| Lesinka | 2013 | Porcelain | 50.1226 | 12.4236 | 49.37 | 0 | 0 | 0 |
| Liba | 2015 | Porcelain | 50.1307 | 12.2328 | 35.79 | 0 | 2 | 2 |
| Loucim | 2010 | Bavaria | 49.3730 | 13.0918 | 62.79 | 0 | 8 | 8 |
| Milhostov | 2013 | Porcelain | 50.1456 | 12.4533 | 51.64 | 0 | 0 | 0 |
| Mnichov | 2013 | Porcelain | 50.0314 | 12.7877 | 75.88 | 0 | 0 | 0 |
| Moedlenreuth | 2014 | Porcelain | 50.1263 | 11.7546 | 2.79 | 0 | 0 | 0 |
| Muehlbach | 2015 | Porcelain | 50.1893 | 12.1659 | 32.27 | 0 | 0 | 0 |
| Muzikov | 2014 | Porcelain | 50.2374 | 12.9502 | 88.13 | 0 | 1 | 1 |
| Nahorecice | 2014 | Porcelain | 50.1488 | 13.2779 | 110.51 | 0 | 1 | 1 |
| Nemcice | 2010 | Bavaria | 49.4276 | 13.0705 | 65.01 | 0 | 1 | 1 |
| Nettles | 2015 | Porcelain | 50.0467 | 12.1462 | 30.21 | 4 | 0 | 4 |
| Neumuehlen | 2010 | Bavaria | 49.2514 | 12.7743 | 36.07 | 0 | 1 | 1 |
| Neumuehlen | 2015 | Bavaria | 49.2514 | 12.7743 | 36.07 | 0 | 2 | 2 |
| Novy Zd’ar | 2015 | Porcelain | 50.2007 | 12.1998 | 34.96 | 0 | 0 | 0 |
| Oberthoelau | 2014 | Porcelain | 50.0341 | 12.0940 | 26.95 | 5 | 0 | 5 |
| Odrava | 2013 | Porcelain | 50.1053 | 12.4792 | 53.31 | 0 | 0 | 0 |
| PerwolfingA | 2015 | Bavaria | 49.2211 | 12.7363 | 31.9 | 0 | 9 | 9 |
| PerwolfingB | 2010 | Bavaria | 49.2283 | 12.7334 | 32.16 | 0 | 4 | 4 |
| Pfaffenthann | 2016 | Bavaria | 49.0782 | 12.3881 | 1.94 | 2 | 0 | 2 |
| Pilgramsreuth | 2014 | Porcelain | 50.2208 | 12.0262 | 24.54 | 4 | 0 | 4 |
| Pleussen | 2015 | Porcelain | 49.9835 | 12.2735 | 40.94 | 0 | 1 | 1 |
| Povodi | 2011 | Porcelain | 50.1351 | 12.4278 | 49.73 | 0 | 0 | 0 |
| Prameny | 2013 | Porcelain | 50.0599 | 12.7258 | 71.16 | 0 | 0 | 0 |
| Pulovice | 2014 | Porcelain | 50.2590 | 12.9578 | 89.08 | 0 | 8 | 8 |
| Radling | 2015 | Bavaria | 49.1813 | 12.6269 | 22.81 | 9 | 0 | 9 |
| Radling Sattel | 2016 | Bavaria | 49.1795 | 12.6341 | 23.14 | 2 | 0 | 2 |
| Rajov | 2013 | Porcelain | 50.0084 | 12.7724 | 75.14 | 0 | 0 | 0 |
| Regenbogen | 2015 | Bavaria | 49.2129 | 12.7100 | 29.8 | 0 | 10 | 10 |
| Roeslau | 2011 | Porcelain | 50.0733 | 12.0063 | 19.81 | 0 | 0 | 0 |
| Rohrbach | 2015 | Porcelain | 50.2339 | 12.3182 | 44.14 | 0 | 2 | 2 |
| Scharlau | 2015 | Bavaria | 49.1912 | 12.6478 | 24.69 | 8 | 0 | 8 |
| Schirnding A | 2015 | Porcelain | 50.0879 | 12.2191 | 34.77 | 0 | 3 | 3 |
| Schoenwald | 2014 | Porcelain | 50.1956 | 12.0939 | 27.64 | 8 | 0 | 8 |
| Semnice | 2014 | Porcelain | 50.241 | 12.9609 | 88.95 | 0 | 0 | 0 |
| Seussen | 2015 | Porcelain | 50.0311 | 12.1618 | 31.71 | 3 | 5 | 8 |
| Spielberg | 2014 | Porcelain | 50.1689 | 12.0396 | 22.98 | 3 | 0 | 3 |
| St Quirin | 2015 | Bavaria | 49.1189 | 12.5319 | 13.35 | 3 | 0 | 3 |
| Stanovice | 2013 | Porcelain | 50.1504 | 12.8738 | 81.66 | 0 | 0 | 0 |
| Stare Sedlo | 2013 | Porcelain | 50.1768 | 12.694 | 69.09 | 0 | 0 | 0 |
| Stary Dvur | 2013 | Porcelain | 50.0874 | 12.8017 | 76.42 | 0 | 0 | 0 |
| Tasching | 2015 | Bavaria | 49.1868 | 12.6699 | 25.8 | 10 | 2 | 12 |
| Teichmuehle | 2016 | Porcelain | 50.0476 | 12.1653 | 31.53 | 2 | 2 | 4 |
| Thiersheim Wasser | 2016 | Porcelain | 50.0709 | 12.1111 | 27.25 | 1 | 0 | 1 |
| Thierstein A | 2011 | Porcelain | 50.0971 | 12.118 | 27.49 | 0 | 0 | 0 |
| Verusicky | 2014 | Porcelain | 50.1355 | 13.1722 | 102.92 | 0 | 13 | 13 |
| Vilzing | 2016 | Bavaria | 49.1822 | 12.6741 | 25.8 | 6 | 0 | 6 |
| Vilzing Field | 2016 | Bavaria | 49.1806 | 12.6817 | 26.2 | 3 | 1 | 4 |
| Vilzing Hanzing | 2016 | Bavaria | 49.1714 | 12.6767 | 25.4 | 3 | 0 | 3 |
| Weissenstaedter Forst | 2011 | Porcelain | 50.0983 | 11.853 | 8.55 | 0 | 0 | 0 |
| Wildenau | 2015 | Porcelain | 50.1993 | 12.1742 | 33.17 | 0 | 0 | 0 |
| Windischbergdorf | 2015 | Bavaria | 49.2421 | 12.7179 | 32.12 | 0 | 2 | 2 |
| Woppmannsdorf | 2010 | Bavaria | 49.1412 | 12.5112 | 13.34 | 0 | 0 | 0 |
| Wunsiedel | 2014 | Porcelain | 50.0464 | 11.9831 | 18.99 | 4 | 0 | 4 |
| Ziegelhuette | 2014 | Porcelain | 49.9979 | 12.0609 | 26.27 | 4 | 0 | 4 |
|  |  |  |  |  |  | 142 | 181 | 323 |

**Supplementary Table S2:** Reference sequences for Tula orthohantavirus (TULV) clades in the order of appearance in the phylogenetic tree (Figure S1) from top to bottom and Puumala orthohantavirus (PUUV) outgroup strains. Columns indicate GenBank accession numbers and TULV clade assignment.

| Accession number | Clade |
| --- | --- |
| NC005224 | PUUV |
| KJ994776 | PUUV |
| Z30942 | Non-European |
| Z30945 | Non-European |
| Z30944 | Non-European |
| Z30941 | Non-European |
| Z30943 | Non-European |
| AM945879 | Non-European |
| AM945877 | Non-European |
| AM945878 | Non-European |
| AF442621 | Non-European |
| KU139528 | Central North |
| KU139531 | Central North |
| KU139529 | Central North |
| KU139530 | Central North |
| KU139533 | Central North |
| DQ662087 | Central North |
| DQ768143 | Central North |
| KU139534 | Central North |
| KU139535 | Central North |
| KU139537 | Central North |
| KU139538 | Central North |
| KU139595 | Central North |
| KU139596 | Central North |
| KU139599 | Central North |
| KU139600 | Central North |
| KU139598 | Central North |
| KU139576 | Central North |
| KU139577 | Central North |
| KU139578 | Central North |
| KU139579 | Central North |
| HQ697346 | Central North |
| HQ697344 | Central North |
| HQ697347 | Central North |
| HQ697351 | Central North |
| EU439948 | Central North |
| EU439950 | Central North |
| EU439946 | Central North |
| EU439951 | Central North |
| EU439949 | Central North |
| EU439947 | Central North |
| EU439952 | Central North |
| GU300137 | Central North |
| GU300136 | Central North |
| DQ662094 | Central North |
| AF063897 | Eastern North |
| AF289820 | Eastern North |
| AF289821 | Eastern North |
| AF289819 | Eastern North |
| KF557547 | Eastern Carpathian |
| Y13979 | Eastern Carpathian |
| Y13980 | Eastern Carpathian |
| AF017659 | Eastern Carpathian |
| AF164093 | Central South |
| HQ697354 | Central South |
| HQ697357 | Central South |
| HQ697353 | Central South |
| HQ697355 | Central South |
| HQ697348 | Central South |
| HQ697349 | Central South |
| HQ697350 | Central South |
| NC005227 | Eastern South |
| Z69991 | Eastern South |
| Z49915 | Eastern South |
| AJ223600 | Eastern South |
| Z48574 | Eastern South |
| Z48741 | Eastern South |
| AJ223601 | Eastern South |
| U95312 | Eastern South |
| KF184327 | Eastern South |
| KF184328 | Eastern South |

**Supplementary Table 3:** Full list of 105 genes 100 kb up- and downstream of a SNP significantly associated with clade specific TULV infections. Chromosome and position show the location of the SNP in the *M. arvalis* reference genome, while distance gives the number of base pairs between the candidate gene and the respective SNP. The p-value refers to the GWAS of all 323 infected individuals.

| Chromosome | Position | Gene | Distance | p-value |
| --- | --- | --- | --- | --- |
| 1 | 4371268 | SOCS3 | 34241 | 2.99E-13 |
| 1 | 4371268 | PGPS1 | 54818 | 2.99E-13 |
| 1 | 4371268 | DYH17 | 90028 | 2.99E-13 |
| 1 | 4414266 | KFA | 17555 | 1.25E-11 |
| 1 | 4414266 | KITH | 50583 | 1.25E-11 |
| 1 | 4414266 | SNG2 | 63136 | 1.25E-11 |
| 1 | 4414266 | TMC8 | 67699 | 1.25E-11 |
| 1 | 4414266 | TMC6 | 95905 | 1.25E-11 |
| 1 | 87846372 | SNG3 | 0 | 1.86E-17 |
| 1 | 87846372 | ZN598 | 5378 | 1.86E-17 |
| 1 | 87846372 | ALR | 7414 | 1.86E-17 |
| 1 | 87846372 | NOXO1 | 12061 | 1.86E-17 |
| 1 | 87846372 | TBL3 | 15444 | 1.86E-17 |
| 1 | 87846372 | RN151 | 26871 | 1.86E-17 |
| 1 | 87846372 | RS2 | 31782 | 1.86E-17 |
| 1 | 87846372 | NDUBA | 33548 | 1.86E-17 |
| 1 | 87846372 | NHRF2 | 37520 | 1.86E-17 |
| 1 | 87846372 | RL3 | 39273 | 1.86E-17 |
| 1 | 87846372 | NTH | 48438 | 1.86E-17 |
| 1 | 87846372 | HS3S6 | 50193 | 1.86E-17 |
| 1 | 87846372 | TSC2 | 55654 | 1.86E-17 |
| 1 | 87846372 | PKD1 | 90093 | 1.86E-17 |
| 2 | 1.73E+08 | RL13 | 0 | 1.50E-15 |
| 2 | 1.73E+08 | OL143 | 4649 | 1.50E-15 |
| 2 | 1.73E+08 | OL147 | 12359 | 1.50E-15 |
| 3 | 95160501 | RFLA | 45063 | 8.48E-14 |
| 3 | 95160501 | NCOR2 | 59845 | 8.48E-14 |
| 3 | 95160501 | ZN664 | 64304 | 8.48E-14 |
| 3 | 1.46E+08 | EI2BE | 0 | 7.59E-16 |
| 3 | 1.46E+08 | DVL3 | 10030 | 7.59E-16 |
| 3 | 1.46E+08 | AP2M1 | 29732 | 7.59E-16 |
| 3 | 1.46E+08 | ABCF3 | 39082 | 7.59E-16 |
| 3 | 1.46E+08 | MRP5 | 53186 | 7.59E-16 |
| 3 | 1.46E+08 | VW5B2 | 70765 | 7.59E-16 |
| 3 | 1.46E+08 | ALG3 | 86221 | 7.59E-16 |
| 3 | 1.46E+08 | EFMT4 | 92273 | 7.59E-16 |
| 4 | 1.94E+08 | USH2A | 0 | 8.65E-16 |
| 5 | 66704092 | SOS1 | 20630 | 6.17E-09 |
| 5 | 66704109 | CDKL4 | 3666 | 1.05E-09 |
| 5 | 66704109 | M4K3 | 59746 | 1.05E-09 |
| 5 | 1.01E+08 | RL38 | 84360 | 5.61E-10 |
| 6 | 2777059 | P5I11 | 34410 | 2.14E-16 |
| 6 | 2777059 | TSN18 | 41717 | 2.14E-16 |
| 6 | 2777059 | ZN431 | 63642 | 2.14E-16 |
| 6 | 21967949 | DPH6 | 0 | 3.56E-12 |
| 6 | 56882593 | NOL4L | 0 | 7.13E-13 |
| 6 | 56882593 | ASXL1 | 58637 | 7.13E-13 |
| 6 | 57051042 | COMD7 | 15273 | 4.03E-17 |
| 6 | 57051042 | DNM3B | 20784 | 4.03E-17 |
| 6 | 57051042 | DNM3C | 53842 | 4.03E-17 |
| 6 | 57051042 | MARE1 | 84074 | 4.03E-17 |
| 6 | 58206488 | IF2B | 53706 | 2.88E-21 |
| 6 | 58206488 | ASIP | 70978 | 2.88E-21 |
| 6 | 58206488 | RALY | 74570 | 2.88E-21 |
| 6 | 58206488 | SAHH | 85231 | 2.88E-21 |
| 6 | 58527535 | PIGU | 0 | 7.66E-20 |
| 6 | 58527535 | MLP3A | 39247 | 7.66E-20 |
| 6 | 58527535 | DLRB1 | 57345 | 7.66E-20 |
| 6 | 58527535 | ITCH | 85038 | 7.66E-20 |
| 6 | 58585061 | T53I2 | 12941 | 1.48E-10 |
| 6 | 58585061 | NCOA6 | 26117 | 1.48E-10 |
| 6 | 58687262 | LORF2 | 12249 | 4.86E-09 |
| 6 | 58687262 | GGT7 | 17172 | 4.86E-09 |
| 6 | 58687262 | ACSA | 47022 | 4.86E-09 |
| 6 | 58839170 | MYH7B | 0 | 3.20E-14 |
| 6 | 58839170 | EDEM2 | 6507 | 3.20E-14 |
| 6 | 58839170 | GSHB | 25604 | 3.20E-14 |
| 6 | 79287623 | NTR1 | 0 | 6.72E-11 |
| 6 | 79287623 | SO4A1 | 39475 | 6.72E-11 |
| 6 | 79287623 | MRGBP | 69602 | 6.72E-11 |
| 6 | 79287623 | OGFR | 79143 | 6.72E-11 |
| 8 | 6747423 | NPT2A | 0 | 3.51E-17 |
| 8 | 6747423 | PROF3 | 11406 | 3.51E-17 |
| 8 | 6747423 | FA12 | 14045 | 3.51E-17 |
| 8 | 6747423 | RGS14 | 18680 | 3.51E-17 |
| 8 | 6747423 | GRK6 | 35557 | 3.51E-17 |
| 8 | 6747423 | LMAN2 | 41520 | 3.51E-17 |
| 8 | 6747423 | PRR7 | 61606 | 3.51E-17 |
| 8 | 6747423 | DREB | 63753 | 3.51E-17 |
| 8 | 6747423 | MAD3 | 78529 | 3.51E-17 |
| 8 | 6747423 | PRLD1 | 82871 | 3.51E-17 |
| 8 | 6747423 | RAB24 | 85972 | 3.51E-17 |
| 8 | 6747423 | PDLI7 | 88222 | 3.51E-17 |
| 8 | 6747423 | NSD1 | 93930 | 3.51E-17 |
| 10 | 32796523 | OSBL1 | 0 | 4.57E-13 |
| 10 | 32796523 | RS15A | 34892 | 4.57E-13 |
| 10 | 32796523 | IMPCT | 91513 | 4.57E-13 |
| 11 | 43643687 | NAKD2 | 0 | 1.11E-11 |
| 11 | 43643687 | RNB3L | 9985 | 1.11E-11 |
| 11 | 43643687 | SKP2 | 39432 | 1.11E-11 |
| 11 | 43643687 | LMBD2 | 73888 | 1.11E-11 |
| 18 | 14516848 | SYC1L | 0 | 1.74E-14 |
| 18 | 14516848 | MON1B | 8498 | 1.74E-14 |
| 18 | 14516848 | ATS18 | 14480 | 1.74E-14 |
| 21 | 26425817 | GDF11 | 0 | 5.68E-11 |
| 21 | 26425817 | BL1S1 | 16714 | 5.68E-11 |
| 21 | 26425817 | SARNP | 20486 | 5.68E-11 |
| 21 | 26425817 | ITA7 | 26931 | 5.68E-11 |
| 21 | 26425817 | MET7B | 50619 | 5.68E-11 |
| 21 | 26425817 | ORML2 | 77305 | 5.68E-11 |
| 21 | 26425817 | DJC14 | 81657 | 5.68E-11 |
| 21 | 26425817 | OLF9 | 81716 | 5.68E-11 |
| 21 | 26425817 | OLF49 | 93511 | 5.68E-11 |
| 21 | 26425817 | TM198 | 95081 | 5.68E-11 |
| 21 | 26425817 | MMP19 | 99059 | 5.68E-11 |
